# Barriers and motivators for *BRCA* genetic testing in Japan

**DOI:** 10.1002/jgc4.70181

**Published:** 2026-02-15

**Authors:** Katsuyuki Konnai, Hiroyuki Fujiwara, Eri Haneda, Ann Sato, Haruya Saji, Hiroto Narimatsu

**Affiliations:** ^1^ Department of Gynecology Kanagawa Cancer Center Yokohama Kanagawa Japan; ^2^ Department of Obstetrics and Gynecology Jichi Medical University Tochigi Japan; ^3^ Department of Genetic Medicine Kanagawa Cancer Center Yokohama Kanagawa Japan; ^4^ Cancer Prevention and Control Division Kanagawa Cancer Center Research Institute Yokohama Kanagawa Japan

**Keywords:** barriers, *BRCA* testing, genetic counseling, insurance, motivator

## Abstract

In Japan, insurance coverage for *BRCA* genetic testing was expanded in 2020. The reasons individuals undergo or decline testing remain unclear; however, cost has been identified as a key factor. Herein, we investigated barriers and motivators for undergoing testing. The subjects were 1025 individuals who received genetic counseling at our institution between April 2014 and March 2024. We included 647 patients and 82 blood relatives. We compared the number of patients undergoing tests before and after insurance coverage to evaluate costs. Patients were classified as tested or not tested. Clinical backgrounds and reasons for undergoing or declining testing were compared retrospectively using medical and counseling records. We found that 485 patients underwent testing. There was a significant increase in the number of patients who underwent testing in the 4 years after coverage (*n* = 376) compared with the 6 years before insurance coverage (*n* = 109; *p* < 0.001). Furthermore, there were significant differences in insurance/self‐pay, treatment status (preoperative/during treatment), and age at first counseling. The main reasons for declining testing were “Discuss options with relatives” (28%) and “Focused on current treatment” (14.3%). Cost‐related concerns markedly decreased after insurance coverage, suggesting cost had been an important structural barrier. Motivators for testing included “Determine the surgical technique” (42.7%) and “For my health care” (22.3%). The blood relative testing rate was high at 91% (75/82). In addition to our finding that cost had been an important barrier to testing, we identified several other non‐medical barriers. In Japan, the selection of candidates for testing and clinical indications are determined according to Japanese HBOC clinical guidelines. We identified determination of the surgical technique as the main motivator, as well as the timing of counseling being important. Understanding barriers and motivators could help clients who need testing.

**What is known about this topic**

Previous studies have reported medical and non‐medical factors that influence *BRCA* genetic testing decisions; however, data from Japan remain limited and have mainly focused on cost as the primary barrier.

**What this paper adds to the topic**

This study demonstrates that, in addition to cost, various non‐medical factors influence testing decisions in Japan, and that expanded insurance coverage has encouraged more proactive, self‐directed decision‐making among patients.

## 1 INTRODUCTION

Hereditary breast and ovarian cancer syndrome (HBOC) is associated with an increased cumulative risk of breast and ovarian cancer (Chen & Parmigiani, 2007). Awareness of HBOC status is important for reducing the risk of ovarian and breast cancer. Risk‐reducing salpingo‐oophorectomy (RRSO) reduces the relative risk of ovarian cancer by about 80% and breast cancer by about 40% among women with *BRCA1/2* mutations who have no history of breast cancer (Domchek et al., 2010; Xiao et al., 2019). In breast cancer patients with pathogenic *BRCA1/2* variants, the risk of developing contralateral breast cancer within 20 years after diagnosis is reported to be 40% for *BRCA1* and 26% for *BRCA2* (Kuchenbaecker et al., 2017). Medical intervention options for the contralateral breast in these patients include surveillance and contralateral risk‐reducing mastectomy (CRRM). CRRM reduces the risk of developing contralateral breast cancer (Jia et al., 2021). For patients currently undergoing cancer diagnosis, *BRCA* status influences decisions regarding surgical technique and PARP inhibitor therapy. On the other hand, for individuals without a cancer diagnosis, identifying pathogenic mutations enables targeted surveillance, cascade testing for at‐risk relatives, and determining the appropriate timing for RRSO or RRM, which are not covered by insurance for individuals without cancer in Japan. Therefore, *BRCA1/2* testing has implications for both cancer prevention and personalized treatment. Diagnosis of HBOC involves genetic counseling followed by *BRCA* genetic testing, with the decision to undergo testing ultimately made by the individual.

Previous studies have reported that individuals with a potential risk for HBOC have both medical and non‐medical reasons for declining to undergo testing (Abdel‐Razeq et al., 2024; Anderson et al., 2012; Armstrong et al., 2000; Hayden et al., 2017; Thompson et al., 2002). Medical reasons included responses such as “Not the best test candidate” and “Not being clinically indicated,” based on the expert judgment of genetic providers. Non‐medical reasons included “Patient disinterest,” “Insurance or out‐of‐pocket cost concerns,” and concerns about “Passing the gene to my children.”

In Japan, we previously reported that lower co‐payments promoted *BRCA* genetic testing (Konnai et al., 2023). However, few studies have investigated factors other than costs. A 2012 study of 40 cases of HBOC found that, in addition to cost, the diagnostic rate not being 100% was a barrier to undergoing testing (Nakata et al., 2012). Furthermore, a nationwide survey on HBOC clinical practice conducted in 2017 identified various reasons including “Resistance to knowing genetic risk,” “Perceived limited benefits of genetic testing,” and “Current lack of desire due to being asymptomatic.” (Komine et al., 2019). However, detailed information was not disclosed.

From July 2018, *BRCA* testing has been partially covered by insurance as a companion diagnostic for olaparib in patients with recurrent breast cancer. Since April 2020, coverage has been expanded to include ovarian cancer, tubal cancer, primary peritoneal cancer, breast cancer patients under 45 years old, triple‐negative breast cancer patients under 60 years old, patients with two or more primary tumors, affected patients with one or more first‐degree relatives with breast or ovarian cancer, and male breast cancer patients. In Japan, genetic testing was historically self‐paid because reimbursement was limited to tests directly linked to treatment decisions. As evidence accumulated demonstrating the clinical utility of *BRCA1/2* testing, including its impact on surgical planning, surveillance, the use of PARP inhibitors, and risk‐reducing surgery, the national health insurance system expanded coverage in 2020. Eligibility for testing is determined according to the Japanese HBOC Clinical Guidelines (Japanese Organization of Hereditary Breast and Ovarian Cancer, 2024), which serve as a domestic equivalent to international criteria such as the NCCN guidelines. These guidelines outline the personal and family histories that warrant *BRCA* testing and are updated regularly as new evidence emerges. The first edition of the Japanese HBOC guidelines was published in 2017, followed by the second edition in 2021 and the third edition in 2024.

Although cost has been identified as a primary barrier to *BRCA* testing in Japan, systematic evaluation of non‐financial barriers remains limited. International studies have identified diverse psychosocial factors, including family dynamics (Li et al., 2018), cancer anxiety (Sun et al., 2020), and cultural beliefs about genetic information (Shaw et al., 2018); however, whether these findings apply to the Japanese cultural context with its emphasis on family harmony (*wa*) and collective decision‐making remains unknown. Additionally, the effect of the expanded insurance coverage that was introduced in April 2020 remains unclear. *BRCA* genetic testing, which is becoming partly covered by insurance in Japan, provides an opportunity to examine the specific effects of cost reduction on patient decision‐making. However, few studies have investigated whether removing financial barriers influences testing decisions. Understanding this insurance‐related behavioral shift may help clarify how cost reduction promotes more informed and autonomous decision‐making regarding genetic testing.

Before the expansion of insurance coverage, *BRCA* genetic testing in Japan was generally available only as a self‐pay service. Kanagawa Cancer Center, as a regional cancer referral hospital, provided a large proportion of these self‐pay tests for patients who sought genetic counseling for HBOC. This study aimed to evaluate the clinical backgrounds and reasons underlying the decision of patients and blood relatives to undergo *BRCA* genetic testing, as well as those who did not undergo testing.

## 2 METHODS

### 2.1 Study design and participants

This was a retrospective observational study conducted at Kanagawa Cancer Center.

The subjects were 874 patients and 151 blood relatives who received genetic counseling between April 2014 and March 2024. The inclusion criterion was patients or blood relatives who received genetic counseling for HBOC. At Kanagawa Cancer Center, genetic counseling is provided regardless of whether genetic testing is ultimately offered or performed. Counseling is conducted for individuals who meet the clinical criteria for HBOC, even if they are not eligible for, decline, or defer genetic testing.

The exclusion criteria were as follows:

1. Individuals who received post‐test genetic counseling after having already undergone multigene panel testing, because their testing decisions were not made within the *BRCA1/2* counseling process.
2. Individuals who received post‐test counseling after participating in a clinical trial for hereditary tumors, because their testing was performed under study‐specific protocols.
3. Individuals who received genetic counseling for family histories of cancers unrelated to HBOC.
4. Individuals who received genetic counseling for hereditary conditions other than HBOC were excluded from the present study because the focus of the present study was to evaluate decision‐making specifically related to *BRCA1/2* testing within the context of HBOC counseling.

Individuals who received *BRCA1/2* testing as part of a multigene panel after genetic counseling were included, provided that *BRCA1/2* analysis was included in the panel.

**FIGURE 1:**
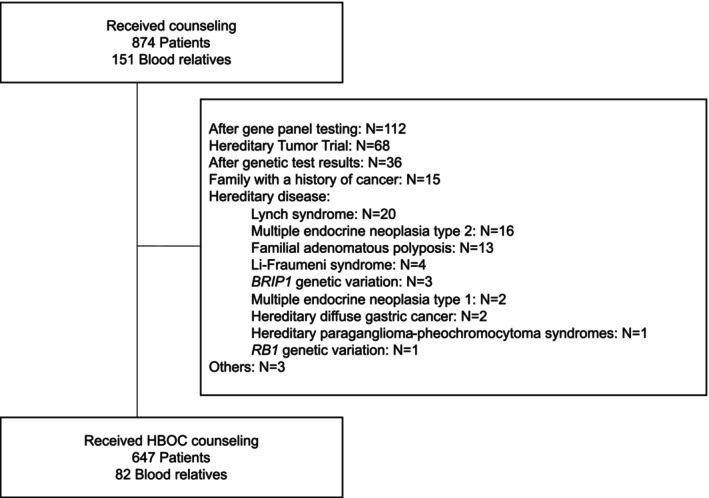
Flowchart of study selection. The subjects were 874 patients and 151 blood relatives who received genetic counseling at Kanagawa Cancer Center between April 2014 and March 2024. We enrolled 647 and 82, respectively. HBOC, hereditary breast and ovarian cancer.

After applying these criteria, 647 patients and 82 blood relatives were included in the final analysis (Figure 1).

### 2.2 Analytical approach

First, to assess the cost impact of *BRCA* genetic testing, we compared the number of tests performed in the pre‐insurance coverage period (from April 2014 to March 2020) with the post‐insurance coverage period (from April 2020 to March 2024).

Next, we divided the patients and blood relatives into two groups: a tested group for cases who underwent *BRCA* genetic testing after genetic counseling, and a not‐tested group. The clinical background of patients and the main reasons for undergoing or not undergoing *BRCA* genetic testing were retrospectively compared between the groups using medical and counseling records. The counseling records were recorded by a certified genetic counselor and were not included in the questionnaire. Patients or relatives were assigned one reason for undergoing *BRCA* genetic testing or for not undergoing it based on their response, following a previous study by Hayden et al. (2017). When multiple reasons were listed, the first one listed was selected. For example, a response of “For my child and for myself” was counted as a response of “For my child.” If no reason was described, it was classified as “unknown.” For patients who did not undergo testing but underwent multiple counseling sessions, the most recent reason for not undergoing testing was used in the analysis.

### 2.3 Study outcomes

The primary outcome of this study was to identify determinants of the decision to undergo or decline *BRCA* genetic testing, including patient‐reported motivators, barriers, and clinical factors associated with testing decisions.

Secondary outcomes included:

1. Trends in *BRCA* test uptake before and after the expansion of insurance coverage.
2. Comparisons of clinical and demographic characteristics between the group that underwent testing and the group that did not.
3. Testing uptake and stated reasons for testing among blood relatives.

### 2.4 Genetic testing procedure

*BRCA1/2* genetic testing was performed at external accredited clinical laboratories. Testing methods included BRACAnalysis® (Myriad Genetics, Salt Lake City, UT, USA) as well as other validated next‐generation sequencing‐based assays, depending on the period and clinical indication. These assays included full sequencing and large genomic rearrangement analysis of *BRCA1* and *BRCA2*. The laboratories reported pathogenic and likely pathogenic variants, as well as variants of uncertain significance (VUS), all of which were included in the present analysis.

### 2.5 Genetic counseling intervention

At the Kanagawa Cancer Center, genetic counseling was provided by certified genetic counselors in collaboration with clinical geneticists or oncologists. All sessions were conducted in person in a private consultation room and included both pre‐ and post‐test counseling when applicable.

In accordance with the Genetic Counseling Intervention Reporting Standards, the counseling sessions included the following components:

1. Indication and risk assessment

Counselors reviewed the patient's personal and family history of breast, ovarian, and other relevant cancers. When possible, a three‐generation pedigree was constructed. Testing eligibility was assessed according to the Japanese HBOC clinical guidelines.

2 Educational content

Counselors explained the purpose of *BRCA1/2* genetic testing; potential test outcomes (pathogenic variant, VUS, or negative); clinical implications for medical management, including risk‐reducing surgery and surveillance options; and the limitations of testing. Institution‐approved printed educational materials were used during the session.

3 Risk communication

Cancer risks were explained using qualitative descriptions and, when appropriate, age‐specific penetrance estimates derived from published literature.

4 Psychosocial counseling

Counselors explored clients' concerns regarding hereditary cancer risk, family communication, and potential psychological burden. Decision‐making support was provided to help clients make informed choices regarding testing.

5 Discussion of testing logistics

Counselors reviewed the testing procedure, cost (self‐pay vs. insurance coverage), expected turnaround time, and possible implications for biological relatives. Clients were informed that they could take additional time to consider their decision and return for follow‐up counseling if needed.

6 Intervention delivery and duration

Sessions were conducted individually and typically lasted approximately 60 min. Most clients received two sessions (one pre‐test and one post‐test), although additional follow‐up sessions were available on request.

7 Provider qualifications

All counseling sessions were conducted by a two‐person team, consisting of a certified genetic counselor and a physician who was either a clinical geneticist or a clinical oncologist. Both were present and participated in each session, ensuring that clients received comprehensive support integrating genetic counseling expertise with medical decision‐making related to hereditary cancer.

This standardized counseling protocol was applied consistently throughout the study period.

### 2.6 Statistical analysis

For the primary analysis, determinants of testing decisions were evaluated by comparing clinical characteristics and reported reasons between the tested and not‐tested groups.

For the secondary analyses, trends in *BRCA* test uptake before and after insurance expansion and patterns among blood relatives were assessed.

The statistical software used was IBM SPSS Version 20 for Windows (IBM, USA). *T*‐test and chi‐square test were used to compare the tested group and not‐tested group. Fisher's exact test was applied when expected cell counts were less than 5. Differences between groups were assessed using analysis of variance followed by Bonferroni post hoc comparisons. For comparisons between the two groups, a two‐sided *p* value of <0.05 was significant.

This study was approved by the Institutional Review Board of Kanagawa Cancer Center (Yokohama, Japan; 2024‐EKI‐71).

## 3 RESULTS

### 3.1 Number of *BRCA* tests and test results

A total of 647 patients and 82 blood relatives received at least one counseling session. Between 2014 and 2024, 486 patients and 75 blood relatives underwent *BRCA* testing, and one patient withdrew consent.

**FIGURE 2:**
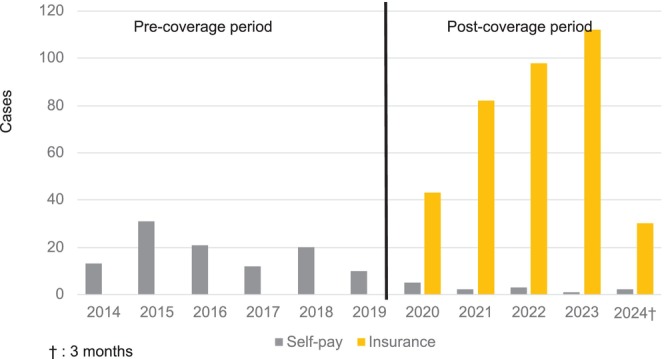
Annual change in *BRCA* testing of patients. Starting in April 2020, insurance coverage has been expanded to include *BRCA* genetic testing and genetic counseling.

In the 6‐year pre‐insurance coverage period (2014–2019), 208 patients (mean: 35/year) received genetic counseling, and 109 (18/year) received *BRCA* testing. In the 4‐year post‐insurance coverage period (2020–2024), 438 patients (110/year) received genetic counseling and 376 (94/year) underwent *BRCA* testing. The rate of testing/counseling significantly increased in the post‐insurance coverage period (52.4% vs. 85.8%; *p* < 0.001; OR = 5.50, 95% CI: 3.9–7.7; Table 1, Figure 2). Notably, eight patients who declined testing during the self‐pay period later underwent *BRCA* testing after insurance coverage became available.

**TABLE 1:** Number of patients who received *BRCA* tests pre‐ and post‐insurance coverage.

	Pre‐coverage period (6 years)	Post‐coverage period (4 years)	*p* ^a^
Number of patients that received genetic counseling (mean)	208 (35/year)	438 (110/year)	
Number of *BRCA* tests (mean)	109 (18/year)	376 (94/year)	
Ratio of testing/counseling	52.4%	85.8%	<0.001

^a^ Chi‐square test.

**FIGURE 3:**
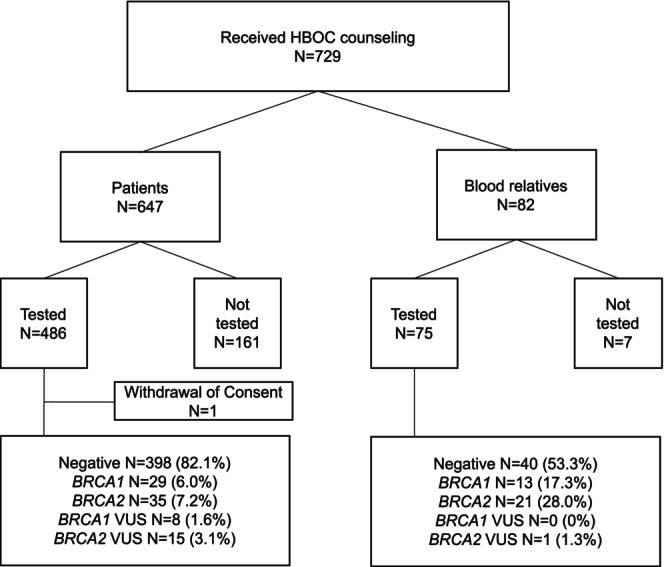
*BRCA* genetic test results. Of 647 patients who received HBOC counseling, 486 had undergone testing. Of 82 blood relatives, 75 had undergone testing. VUS, variant of uncertain significance.

Pathogenic variants were identified in *BRCA1* in 29 patients (6.0%) and *BRCA2* in 35 patients (7.2%), whereas among blood relatives, *BRCA1* and *BRCA2* pathogenic variants were found in 13 (17.3%) and 21 (28.0%), respectively (Figure 3).

### 3.2 Clinical background of patients

Table 2 shows the patients' clinical backgrounds. The age at first counseling was significantly younger in the tested group (52 years old) than in the not‐tested group (55 years old; *p* = 0.01). With respect to treatment status, preoperative status was significantly more commonly cited as a reason for undergoing *BRCA* testing compared with status during treatment (*p* < 0.001). In the tested group, more patients used insurance than self‐paid (*p* < 0.001). There were no significant differences in sex, cancer type, hormone sensitivity type, incidence of bilateral breast cancer, family history, parity, referring physician, and counseling companion between the two groups (Table 2).

**TABLE 2:** Clinical background of patients.

	Tested, *N* = 485	Not tested, *N* = 161	*p*
Age at first counseling, mean (SD)	52 (12.2)	55 (13.2)	0.01^a^
Sex			0.66^b^
Female	473	156	
Male	12	5	
Cancer type			0.65^c^
Breast	456	149	
Ovarian	22	8	
Other	7	4	
Hormone sensitivity type (breast cancer)			0.23^c^
Luminal (%)	336 (73.7)	115 (77.1)	
Triple‐negative (%)	69 (15.1)	26 (17.4)	
HER2 (%)	45 (9.9)	6 (4.0)	
Unknown (%)	6 (1.3)	2 (1.3)	
Bilateral (breast cancer)			0.67^b^
Metachronous	64	22	
Synchronous	44	11	
Family history			0.14^c^
Yes	345	127	
No	139	34	
Unknown	1	0	
Parity			0.58^b^
Yes	360	123	
No	125	38	
Treatment status			<0.001^c^, ^d^
Preoperative (a)	283	67	
Postoperative (b)	104	39	
During treatment (c)	44	32	
After treatment (d)	54	23	
Referrer			0.07^b^
Physician	355	106	
Patient's request	130	55	
Counseling companion			0.49^b^
Without	308	107	
With	177	54	
Insurance/Self‐pay			<0.001^b^
Insurance	365	59	
Self‐pay	120	102	

^a^ *T*‐test.

^b^ Chi‐square test.

^c^ ANOVA with Bonferroni correction.

^d^ a versus c, *p* < 0.01.

### 3.3 Main reasons for patients not undergoing *BRCA* testing

Of the 647 patients, 161 did not undergo testing. The most common reason for not undergoing testing was “Discuss options with relatives,” followed by “Focused on current treatment,” “Impact on blood relatives,” “Fewer benefits,” and “Resistance to knowing genetic risk (self).” Before the April 2020 expansion of insurance coverage, 99 patients did not undergo testing, whereas after the expansion, 62 patients did not undergo testing. Of the 62 patients who did not undergo testing after coverage was expanded, 59 were eligible for insurance coverage but did not receive it. The reasons for not undergoing the test varied; however, no patients reported medical reasons during the entire study period. During the post‐coverage period, there were significant decreases in “Cost concerns” (*p* = 0.03) and “Unknown” (*p* = 0.04) responses (Table 3).

**TABLE 3:** Main reasons for patients not undergoing *BRCA* testing.

Reason	Number (%)	Pre‐coverage period, number (%)	Post‐coverage period, number (%)	*p* ^a^
Discuss options with relatives	45 (28.0)	24 (24.2)	21 (33.9)	0.21
Focused on current treatment	23 (14.3)	16 (16.1)	7 (11.3)	0.49
Impact on blood relatives	21 (13.0)	11 (11.1)	10 (16.1)	0.35
Fewer benefits	18 (11.2)	9 (9.1)	9 (14.5)	0.31
Resistance to knowing genetic risk (self)	15 (9.3)	10 (10.1)	5 (8.0)	0.78
Cost concerns	12 (7.5)	11 (11.1)	1 (1.6)^b^	0.03
Low risk assessment (self, blood relatives)	4 (2.5)	3 (3.0)	1 (1.6)	1.00
Indifferent	4 (2.5)	1 (1.0)	3 (4.8)	0.16
Concerns about surveillance or risk‐reduction surgery	3 (1.8)	1 (1.0)	2 (3.2)	0.56
Opposition by blood relatives	2 (1.2)	0 (0)	2 (3.2)	0.64
Other reason	7 (4.3)	6 (6.1)	1 (1.6)	0.25
Unknown	7 (4.3)	7 (7.1)	0 (0)	0.04
Total	161	99	62	

^a^ Fisher's exact test.

^b^ Breast cancer patients not covered by insurance.

### 3.4 Main reasons for patients undergoing *BRCA* testing

Of the 647 patients, 486 underwent testing. One patient withdrew consent after testing; therefore, we included 485 patients. In the combined pre‐ and post‐insurance coverage periods, the main reason was “Determine the surgical technique or treatment plan,” followed by “For my health care or future,” “For blood relatives,” and “For insurance or High‐cost Medical Expense Benefit coverage.” The motivator “For insurance or High‐cost Medical Expense Benefit coverage” referred to two situations: (1) patients who chose testing because *BRCA* testing became newly covered by insurance, and (2) patients who chose to reduce their out‐of‐pocket costs by undergoing testing in the same month as other medical treatments due to Japan's monthly out‐of‐pocket maximum under the High‐cost Medical Expense Benefit system. Compared with the pre‐coverage period, there were significant increases in the post‐coverage period in the responses “For my health care or future” (*p* < 0.001) and “For insurance or High‐cost Medical Expense Benefit coverage” (*p* < 0.001), whereas there were significant decreases in “Recommended by a blood relative” (*p* = 0.01) and “Blood relatives have developed cancer (including previous cases)” (*p* = 0.03; Table 4).

**TABLE 4:** Main reasons for patients undergoing *BRCA* testing.

Reason	Number (%)	Pre‐coverage period, number (%)	Post‐coverage period, number (%)	*p* ^a^
Determine the surgical technique or treatment plan	207 (42.7)	46 (42.2)	161 (42.8)	1.00
For my health care or future	108 (22.3)	11 (10.1)	97 (25.8)	<0.001
For blood relatives	99 (20.4)	23 (21.1)	76 (20.2)	0.89
For insurance or High‐cost Medical Expense Benefit coverage	22 (4.5)	0 (0)	22 (5.9)	<0.001
Recommended by a blood relative	10 (2.1)	6 (5.5)	4 (1.1)	0.01
Blood relatives have developed cancer (including previous cases)	9 (1.9)	7 (6.4)	2 (0.5)	0.03
Worried about ovarian or pancreatic cancer	9 (1.9)	5 (4.6)	4 (1.1)	0.16
Recommended by the doctor	3 (0.6)	0 (0)	3 (0.8)	0.24
Want to receive risk‐reducing surgery	3 (0.6)	2 (1.8)	1 (0.2)	0.23
High‐risk assessment (self, blood relatives)	2 (0.4)	2 (1.8)	0 (0)	0.11
Ovarian tumor found (including pre‐existing)	2 (0.4)	2 (1.8)	0 (0)	0.11
Anxious not to know	2 (0.4)	2 (1.8)	0 (0)	0.11
Other reason	3 (0.6)	2 (1.8)	1 (0.2)	0.23
Unknown	6 (1.2)	1 (0.9)	5 (1.3)	0.46
Total	485	109	376	

^a^ Fisher's exact test.

### 3.5 Main reasons for blood relatives undergoing *BRCA* testing

Of 82 blood relatives, 75 underwent testing. In this study, testing of relatives included both cascade testing in families with a known pathogenic *BRCA* variant and testing of at‐risk relatives without an identified familial mutation. Among the 75 blood relatives who underwent *BRCA* testing, 69 (92.0%) received testing as cascade testing following the identification of a pathogenic *BRCA* variant in a family member, whereas 6 (8.0%) underwent testing without a previously identified familial variant. The main reason for undergoing testing was “For my health care or future,” followed by “For blood relatives.” These two reasons accounted for about 90% of responses. “Want to receive risk‐reducing surgery” was also given as a reason by 2.7% (Table 5).

**TABLE 5:** Main reasons for blood relatives undergoing *BRCA* testing after receiving genetic counseling.

Reason	Number (%)
For my health care or future	61 (81.3)
For blood relatives	7 (9.3)
Recommended by a blood relative	2 (2.7)
Want to receive risk‐reducing surgery	2 (2.7)
Worried about ovarian or pancreatic cancer	1 (1.3)
Ovarian tumor found (including pre‐existing)	1 (1.3)
Unknown	1 (1.3)
Total	75

## 4 DISCUSSION

In the present study, we identified barriers and motivators for *BRCA* genetic testing in Japan. A previous study reported that lower co‐payments promote *BRCA* genetic testing and risk‐reducing salpingo‐oophorectomy (Konnai et al., 2023). Our previous study found that the greatest barrier was the cost of testing (Konnai et al., 2023). We investigated the number of people undergoing *BRCA* genetic tests over a 10‐year period from 2014. For the first 6 years, all counseling and genetic tests were entirely self‐paid; however, during the latter 4 years, insurance coverage for genetic testing was expanded to include *BRCA* testing. Regarding reasons for not undergoing testing, “Cost concerns” significantly decrease during the post‐coverage period. Since 2020, genetic testing covered by insurance has increased significantly. In Japan, the cost of *BRCA* genetic testing is approximately ¥200,000 (US$1360 [1 USD = ¥147]), resulting in a patient payment of about ¥60,000 (US$408) under the standard 30% co‐payment rate. Eight patients did not undergo the test at self‐pay but chose to undergo testing only after insurance coverage became available. This pattern demonstrates that financial barriers had a direct and meaningful impact on testing decisions, as some individuals reconsidered and ultimately chose testing only after the out‐of‐pocket cost was reduced. These findings provide real‐world evidence that lowering the financial burden improves uptake of genetic testing, which supports the findings of previous reports from other countries.

The importance of financial accessibility has been reported worldwide. Previous systematic reviews identified cost, privacy or confidentiality, and psychological harm as major determinants of testing decisions (Smith‐Uffen et al., 2021; Sweeny et al., 2014). A Korean study on national insurance expansion reported that *BRCA* testing programs were more likely to be successfully implemented when supported by financial coverage, insurance‐based reimbursement, and integration with genetic counseling services (Jang et al., 2024). These findings are consistent with our results that indicate that reducing co‐payments leads to more proactive and voluntary decision‐making.

In Japan, barriers other than cost were diverse. The main reason was “Discuss options with relatives,” followed by “Focused on current treatment,” “Impact on blood relatives,” “Fewer benefits,” and “Resistance to knowing genetic risk (self).” These findings are similar to previous studies (Armstrong et al., 2000; Godard et al., 2007; Hayden et al., 2017; Peterson et al., 2002; Thompson et al., 2002) that reported “Cost concerns,” “Impact on blood relatives,” and “Patient indifference” as common reasons. In many Asian populations, decisions regarding genetic testing are influenced by collective family values and interdependence rather than individual autonomy (Glenn et al., 2012; Li et al., 2018; Shaw et al., 2018; Sun et al., 2020). These studies reported that emotional restraint, family harmony, and the desire to protect relatives from distress often shape decision‐making. Our findings are consistent with these reports, as we identified “Discuss options with relatives” as a major reason for declining testing, even after testing became covered by insurance. On the other hand, medical reasons, such as “Not the best test candidate” or “Not clinically indicated,” as well as “Discrimination or confidentiality concerns,” were not identified as factors. This suggests that the selection of candidates for testing and clinical indications is appropriate in Japan, although various non‐medical barriers remain.

Motivators, other than cost and surgical technique, included “For my health care or future,” “For blood relatives,” and “Recommended by a blood relative,” in that order. Comparing pre‐ and post‐insurance coverage periods, “For my health care or future” responses significantly increased in the post‐coverage period. On the other hand, “Recommended by a blood relative” and “Blood relatives have developed cancer (including previous cases)” responses significantly decreased. These shifts suggest that patients are increasingly making testing decisions based on their own judgments rather than external influences such as recommendations from relatives. Furthermore, as genetic testing has become more widely available due to it becoming covered by insurance, patients may have made decisions to undergo testing based on information they obtained themselves. Over time, the importance of genetic testing has become more widely acknowledged not only among healthcare providers but also among patients and their families, facilitating more autonomous decision‐making, which may explain the increases in the number of tests every year since 2020. D'Andrea et al. (2016) reported that a reduction in testing costs can substantially improve the cost‐effectiveness of implementation strategies, underscoring the important role of affordability in the successful delivery of genetic testing. Our finding that patient participation increased substantially once testing fees were covered by national insurance and financial barriers were largely removed provides real‐world evidence from Japan supporting these previous studies. Thus, cost reduction had both an economic and behavioral impact.

In addition to the increased number of tests, the proportion of *BRCA1/2*‐positive results remained stable before and after the expansion of insurance coverage. Among patients, the positivity rate was approximately 13% in both periods (14/109 before vs. 50/376 after coverage expansion), and among blood relatives it remained high at around 45–50% (7/14 vs. 27/61). The stability of positivity rates, despite a substantial increase in testing volume, indicates that *BRCA* testing has continued to be offered to an appropriately selected high‐risk population rather than being extended indiscriminately. Our findings support the appropriateness of the clinical criteria used to identify candidates for *BRCA* testing in Japan.

Problems with genetic counseling were also identified. Of the 161 patients who did not undergo genetic testing, 159 made their decision after a one‐time counseling session. Although many respondents replied that they planned to “Discuss options with relatives,” our findings suggest that this did not occur. A similar finding was reported in a previous study (McCarthy, 2019). Additionally, there were several cases where patients expressed their intention to undergo testing during counseling; however, when the staff advised them to consider their decision at home, they subsequently chose not to undergo testing. This finding does not imply that successful genetic counseling requires patients to undergo testing, as declining testing can also reflect an exercise of patient autonomy. Thus, the decision to decline testing should be understood as a valid outcome of nondirective counseling rather than as a failure of counseling. Some patients may need additional opportunities to revisit information or concerns before making an autonomous decision. In Japan, decisions about genetic testing are often influenced by discussions with family members; thus, structured follow‐up after the initial counseling session may be useful. Our findings suggest that a follow‐up approach is necessary. Although some patients said they would discuss the decision with their relatives, no further counseling sessions were held; therefore, their subsequent decision‐making process could not be assessed. In our institution, genetic counseling is primarily conducted in person, and formal follow‐up counseling by telephone is not routinely provided. Although family members who live in distant areas may join the session remotely via FaceTime or Zoom, patients themselves do not typically choose to receive counseling through remote platforms. As a result, when patients defer their decision after a single session, opportunities for timely follow‐up are limited. Therefore, alternative platforms could help supplement in‐person services and provide more flexible follow‐up options. As an improvement strategy, incorporating digital tools, such as telemedicine platforms, online counseling systems, and AI‐based chatbots (Pederson & Narod, 2024), could potentially supplement limited counseling resources and provide patients with more accessible, personalized support.

Although blood relatives in Japan must pay the full cost of *BRCA* testing out of pocket, the uptake rate in our cohort remained remarkably high at 91%. This high uptake rate among blood relatives largely reflects cascade testing following the identification of a pathogenic *BRCA* variant in a family member, with more than 90% of tested relatives undergoing testing for this reason. This contrasts with systems in other countries, such as the United States, where free cascade testing programs are available for relatives of individuals with pathogenic variants. The high uptake observed in our study may reflect strong motivation, the influence of probands who had already received a positive result, and the perceived personal utility of testing among relatives. Further studies are needed to investigate whether the introduction of publicly funded or reduced‐cost cascade testing in Japan increases uptake, particularly among younger or more distant relatives. Future policy discussions are needed to consider the use of family‐based financial support models to reduce disparities and promote more equitable access to genetic testing.

In the present study, the majority of probands, such as parents or siblings, of blood relatives were identified as having HBOC (75 out of 82). The age at first counseling was 38 years in the tested group, which is younger than the 48 years in the non‐tested group (*p* = 0.03). These findings suggest a positive attitude among probands to make active use of genetic test results, “For my health care or future.”

Although our study did not directly evaluate access to genetic counseling or *BRCA* testing, access‐related disparities have been reported in previous studies that were conducted across Asia. Nakamura et al. (2016) reviewed HBOC management in Asian countries and noted that differences in public awareness and insurance systems contribute to unequal access to genetic services. Jang et al. (2024) similarly reported that Korea's insurance expansion and the integration of genetic counselors significantly increased *BRCA* testing uptake. These findings suggest that structural factors, such as national insurance policies and the availability of specialized counseling infrastructure, influence testing uptake. Further research is needed to examine how such access‐related factors operate within Japan and how they may affect decision‐making among individuals at risk for HBOC.

This study has several limitations and strengths. First, because the participants were all patients who received genetic counseling at a cancer center, selection bias cannot be excluded as these individuals may have higher awareness of hereditary cancer and stronger motivation to undergo testing compared with the general population. Therefore, our findings may not be fully generalizable to patients in other clinical settings. Second, previous studies have reported that disparities in education and income are related to undergoing *BRCA* testing (Cragun et al., 2015; Olaya et al., 2009). However, in the present study, counseling records did not include information on educational background or income; therefore, these factors could not be evaluated. Third, the reasons for undergoing or not undergoing testing were unclear in some cases because they were based on counseling and medical records; however, the number of these cases was small. The strengths of our study include the long observation period from the time of expanded insurance coverage and the relatively large sample size (729 cases) compared with previous studies.

## 5 CONCLUSIONS

We identified several barriers and motivators for undergoing *BRCA* genetic testing in Japan. Although cost was not the most frequently stated reason for declining testing, our findings indicate that it had previously functioned as a major structural barrier, as evidenced by the marked reduction in cost‐related concerns after coverage was expanded. With the expansion of insurance coverage, this issue is being progressively resolved. Our long‐term analysis demonstrated that reducing co‐payments significantly increased screening rates and promoted more autonomous and voluntary decision‐making. We identified the determination of the surgical technique as the main motivator and found that the timing of counseling is important. In Japan, the selection of candidates for testing and clinical indications is appropriate. If patients do not undergo testing during the first counseling, repeated counseling may be necessary. Repeated counseling sessions may support patients in clarifying their values and concerns, rather than directing them toward testing. In the Japanese context, decisions about genetic testing often involve discussions with family members, which can take time. Therefore, opportunities for follow‐up counseling may help patients reach an autonomous and well‐informed decision without compromising the nondirective nature of genetic counseling. Understanding barriers and motivators could help encourage patients to undergo testing.

### 5.1 Implications for genetic counseling

The findings of this study highlight several important considerations for genetic counseling practice in the context of HBOC in Japan. First, although the expansion of insurance coverage substantially increased testing uptake, cost‐related barriers persisted for some individuals, particularly for blood relatives who must still pay out of pocket. Counselors should therefore assess financial concerns directly and provide clear guidance on available support resources. Policy‐level changes, such as reduced‐cost cascade testing, may further promote equitable access.

Second, “Discuss options with relatives” was one of the most common reasons for declining testing, underscoring the central role of family communication in decision‐making. Genetic counselors should anticipate this cultural dynamic and offer structured follow‐up opportunities rather than relying solely on a single‐session model. Providing scheduled follow‐up, telemedicine options, or digital decision‐support tools may help patients revisit information and make autonomous choices after family discussions.

Third, our findings suggest that many patients undergo *BRCA* testing to guide surgical planning or future medical management. Timely counseling that aligns with the clinical decision‐making timeline is therefore essential. Coordination between clinical teams and genetic counseling services remains critical to ensure that patients receive counseling early enough to integrate results into treatment planning.

Finally, the high uptake among blood relatives even under self‐pay conditions indicates a strong perceived personal utility of testing. Clear communication about the benefits of cascade testing and personalization of risk information may further encourage uptake by at‐risk relatives.

Overall, genetic counseling for HBOC should incorporate culturally aware communication strategies, proactive follow‐up systems, and timely intervention to support informed and autonomous decision‐making in the Japanese healthcare context.

## AUTHOR CONTRIBUTIONS

KK and HF conceptualized and designed the study. KK and HF analyzed and interpreted the data. KK, HF, and HN drafted the manuscript. KK, HF, and HN performed statistical analysis. KK, EH, AS, and HN provided genetic counseling. HF and HS supervised the project. All authors read and approved the final manuscript.

## FUNDING INFORMATION

This research received no specific grant from any funding agency.

## CONFLICT OF INTEREST STATEMENT

All authors have no conflict of interest relevant to this study.

## ETHICS STATEMENT

Human studies and informed consent: This study was submitted and approved by the Institutional Review Board of Kanagawa Cancer Center (Yokohama, Japan; 2024‐EKI‐71). This study was carried out following the Declaration of Helsinki.

Animal studies: No nonhuman animal studies were carried out by the authors for this article.

## PATIENT CONSENT STATEMENT

This study was conducted retrospectively using anonymized clinical and counseling records. In accordance with institutional policy and national regulations, the requirement for written informed consent was waived.

## Data Availability

The data that support the findings of this study are available from the corresponding author upon reasonable request.
